# Biopsy with Ureterorenoscopy Before Radical Nephroureterectomy is Associated with Increased Intravesical Recurrence in Urothelial Cancer Located in the Kidney

**DOI:** 10.5152/tud.2022.22143

**Published:** 2022-11-01

**Authors:** Meftun Culpan, Mehmet Caglar Cakici, Ferhat Keser, Mehmet Yigit Yalcin, Taner Kargi, Rıdvan Kayar, Erdal Abay, Gorkem Ozenc, Ali Kumcu, Mehmet Pehlivanoglu, Semih Turk, Erdem Kisa, Selcuk Sahin, Metin Ishak Ozturk, Alper Otunctemur, Resul Sobay, Huseyin Cihan Demirel, Omer Yilmaz, Gokhan Atis, Muhammet Abdurrahim Imamoglu, Asif Yildirim

**Affiliations:** 1 Department of Urology, İstanbul Medeniyet University Faculty of Medicine, İstanbul, Turkey; 2 Department of Urology, İstanbul Göztepe Prof. Dr. Süleyman Yalçın City Hospital, İstanbul, Turkey; 3 Department of Urology, University of Health Sciences, İzmir Tepecik Training and Research Hospital, İzmir, Turkey; 4 Department of Urology, University of Health Sciences, Bakirkoy Sadi Konuk Training and Research Hospital, İstanbul, Turkey; 5 Department of Urology, University of Health Sciences, Haydarpasa Numune Training and Research Hospital, İstanbul, Turkey; 6 Department of Urology, University of Health Sciences, Prof. Dr. Cemil Taşçıoğulu City Hospital, İstanbul, Turkey; 7 Department of Urology, University of Health Sciences, Diskapi Yıldırım Beyazıt Training and Research Hospital, İstanbul, Turkey; 8 Department of Urology, University of Health Sciences, Ümraniye Training and Research Hospital, İstanbul, Turkey; 9 Department of Urology, University of Health Sciences, Sultan 2. Abdulhamid Han Training and Research Hospital, İstanbul, Turkey; 10 Department of Urology, University of Health Sciences, Şişli Hamidiye Etfal Training and Research Hospital, İstanbul, Turkey; 11 Department of Urology, University of Health Sciences, Haydarpaşa Training and Research Hospital, İstanbul, Turkey

**Keywords:** Biopsy, intravesical recurrence, radical nephroureterectomy, survival, upper urinary tract carcinoma, ureterorenoscopy

## Abstract

**Objective:**

Diagnostic ureterorenoscopy is used to identify upper tract urothelial cancer before radical nephroureterectomy, especially for uncertain lesions in imaging modalities or urine cytology. However, diagnostic ureterorenoscopy can potentially cause intravesical tumor spillage and can increase intravesical recurrence rates. We aimed to investigate the impact of diagnostic ureterorenoscopy before radical nephroureterectomy, with and without biopsy, on intravesical recurrence rates of patients with upper tract urothelial cancer.

**Material and methods:**

Patients with localized upper tract urothelial cancer from 8 different tertiary referral centers, who underwent radical nephroureterectomy between 2001 and 2020, were included. Three groups were made: no URS (group 1); diagnostic ureterorenoscopy without biopsy (group 2); and diagnostic ureterorenoscopy with biopsy (group 3). Intravesical recurrence rates and survival outcomes were compared. Univariate and multivariate Cox regression analyses were performed to determine the factors that were associated with intravesical recurrence-free survival.

**Results:**

Twenty-two (20.8%), 10 (24.4%), and 23 (39%) patients experienced intravesical recurrence in groups 1, 2, and 3, respectively (*P* = .037) among 206 patients. The 2-year intravesical recurrence-free survival rate was 83.1%, 82.4%, and 69.2%, for groups 1, 2, and 3, respectively (*P* = .004). Cancer-specific survival and overall survival were comparable (*P* = .560 and *P* = .803, respectively). Diagnostic ureterorenoscopy + biopsy (hazard ratio: 6.88, 95% CI: 2.41-19.65, *P* < .001) was the only independent predictor of intravesical recurrence in patients with upper tract urothelial cancer located in the kidney, according to tumor location.

**Conclusion:**

Diagnostic ureterorenoscopy + biopsy before radical nephroureterectomy significantly increased the rates of intravesical recurrence in tumors located in kidney. This result suggests tumor spillage with this type of biopsy, so further studies with different biopsy options or without biopsy can be designed.

**Main Points**

- Intravesical recurrence (IVR) is a common problem after radical nephroureterectomy (RNU) and can cause serious morbidity.
- Free circulating tumor cells in urine can implant to the damaged bladder mucosa and cause IVR.
- In patients with upper tract urothelial cancer, especially located in kidney, diagnostic ureterorenoscopy + biopsy before RNU was substantially related with higher IVR.

## Introduction

Upper urinary tract urothelial cancers (UTUCs) are infrequent and constitute 5%-10% of all urothelial cancers. However, due to improved detection methods and higher bladder cancer survival rates, the incidence rate has climbed in recent years.^1^ Radical nephroureterectomy (RNU) is the preferred treatment option for most localized UTUCs.^2^ Despite this aggressive treatment, approximately 30% of patients experience disease relapse, especially intravesical recurrence (IVR).^3,4^ Intravesical recurrence can reduce patients’ quality of life because of cystoscopic surveillance and recurrent transurethral resections of bladder tumor operations or can cause disease progression.

Diagnostic ureterorenoscopy (d-URS) is commonly used to identify UTUCs before RNU, especially for uncertain lesions in imaging modalities or urine cytology. However, d-URS can potentially cause intravesical tumor spillage, and because of this IVR rates may increase after d-URS. The impact of d-URS before RNU was first investigated by Hendin et al.^5^ and the authors concluded that d-URS had no negative effects on recurrence rates or cancer-specific survival (CSS). After that, limited studies have been published on this issue; however, there have been conflicting results among the studies.^6-8^ Finally, in a recent study, it has been shown that ureterorenoscopy (URS) with biopsy was associated with increased IVR, but URS without biopsy was not.^9^

We hypothesized that d-URS with biopsy (d-URS + bx) can increase IVR rates because of increased tumor spillage. We aim to examine the effects of d-URS before RNU, with and without biopsy, on the IVR rates of patients with UTUCs. As a secondary analysis, we also studied the cancer-specific and overall survival (OS) of these patients.

## Materials and Methods

**Figure 1.**
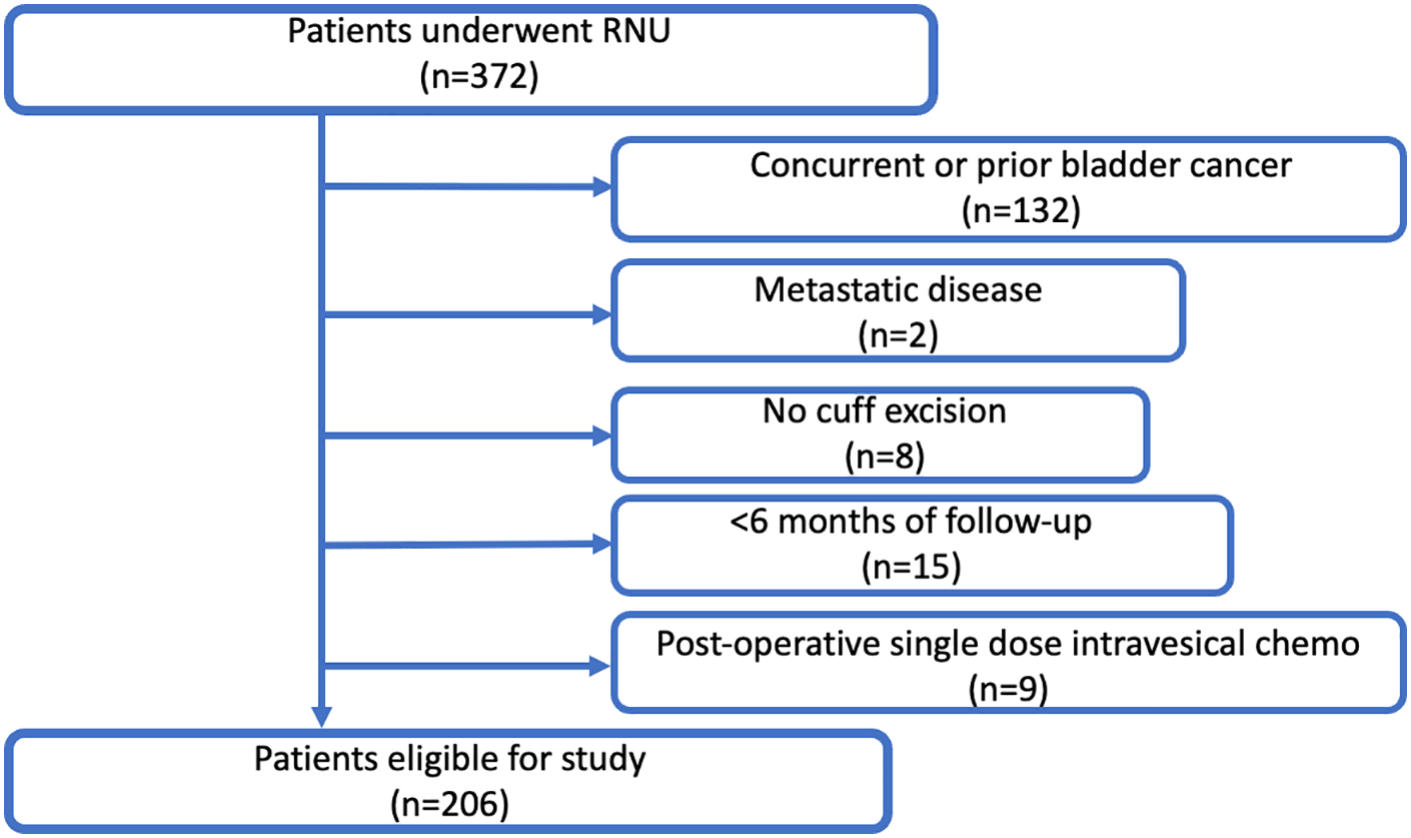
Study flowchart for inclusion criteria. RNU: radical nephroureterectomy.

This multicenter study was conducted retrospectively after local ethics committee approval (decision no: 2021/0125, decision date: February 10, 2021). Patients from 8 different tertiary referral centers who underwent RNU and had localized UTUC between 2001 and 2020 were included in our study. Diagnostic cystoscopy was performed in all patients just prior to RNU to exclude concurrent bladder cancer. Our exclusion criteria were presence of concurrent or prior bladder cancer (n = 132), metastatic disease (n = 2), absence of bladder cuff excision (n = 8), and less than 6 months of follow-up (n = 15). Patients with postoperative single-dose intravesical chemotherapy (n = 9) were also excluded to avoid possible effects of this variable on IVR (Figure 1). In addition to this, the number of patients with postoperative single-dose intravesical chemotherapy was very small for statistical analysis. Patients’ demographics, tumor characteristics, and follow-up variables were evaluated retrospectively.

Informed consent was obtained from all individual participants included in the study.

Patients were assessed with computerized tomography urography (CTU) or magnetic resonance imaging urography (MRU) for diagnosis of UTUC and metastasis. After diagnostic cystoscopy, RNU operations were performed with open, laparoscopic, or robotic techniques, and all patients underwent open bladder cuff excision with an extravesical approach. Intramural ureters were excised with partial cystectomy with negative surgical margin. If there was suspicion of lymph node involvement in imaging modalities prior to RNU, a lymphadenectomy was performed. The time from first diagnosis with imaging techniques to RNU was defined as diagnosis treatment period. After RNU, patient follow-up was performed with regular cystoscopy, urine cytology (quarterly for the first 2 years, every 6 months for the next 3 years, and then annually), and imaging modalities (every 6 months for 2 years and then annually, with CTU or MRU). Diagnosis of biopsy-proven bladder cancer at follow-up was defined as IVR. Pathological specimens were assessed according to the 2004 WHO classifications for tumor grade and tumor–node–metastasis stage at each institution where the RNU was performed. Tumor size was determined by the pathological examination.

The investigations were carried out by dividing the patients into 3 groups. Group 1 included patients who underwent RNU according to imaging modalities and/or urine cytology without d-URS (no URS). Group 2 included patients who underwent RNU with preoperative d-URS without biopsy (d-URS). Group 3 consisted of patients who underwent RNU with preoperative d-URS + bx. Our primary end point was the determination of IVR during follow-up. Intravesical recurrence rates were compared between the 3 groups, and factors predicting IVR were examined. The secondary end points were CSS and OS. Time from the date of the RNU to the last visit or death was defined as the follow-up period.

### Statistical Analysis

For quantitative data, the 1-sample Kolmogorov–Smirnov test was performed to evaluate the normality of the distribution. Quantitative variables with normal distribution were specified as mean ± SD, and variables without normal distribution were presented as median (range) for descriptive statistics in the study. The 1-way analysis of variance was applied for the quantitative variables that had a normal distribution, and the Kruskal–Wallis test was applied for the others. Pairwise comparisons were analyzed with the Student’s *t*-test for the quantitative variables that had a normal distribution, and the Mann–Whitney *U*-test was applied for the others. A comparison of independent categorical variables was performed by a Pearson chi-square test and Fisher’s exact test. Cumulative survival percentages were calculated using the Kaplan–Meier method, and the significance of differences in the survival rate was analyzed using the log-rank test. Univariate and multivariate Cox proportional hazards regressions were performed to determine the factors that were associated with IVR-free survival (IV-RFS). Those with a *P*-value of <.2 in univariate analysis were included in multivariate analysis. All statistical analyses were performed using the Statistical Package for the Social Sciences for Windows version 22.0 (IBM SPSS Corp.; Armonk, NY, USA) where the probability of alpha error was assumed to be *α* = 0.05.

## Results

After exclusions, 206 patients were included with 24 months of follow-up. The median age was 66 years, and 24.75% of the patients were female. The no URS, d-URS, and d-URS + bx groups consisted of 106, 41, and 59 patients, respectively. Patients’ demographics, clinicopathologic features, and follow-up statuses were compared between the 3 groups and are demonstrated in Table 1. Multifocal tumor rates were significantly lower in group 1 than in groups 2 and 3 (13.2%, 34.1%, and 25.4%, respectively, *P* = .012). Median tumor size was significantly higher in group 1 than in groups 2 and 3 (50 mm, 40 mm, and 38 mm, respectively, *P* = .001), and there was a statistically significant difference between the 3 groups according to the location of the UTUC (*P* = .013). Diagnosis treatment period was significantly longer in group 3 (*P* = .001).

**Figure 2.**
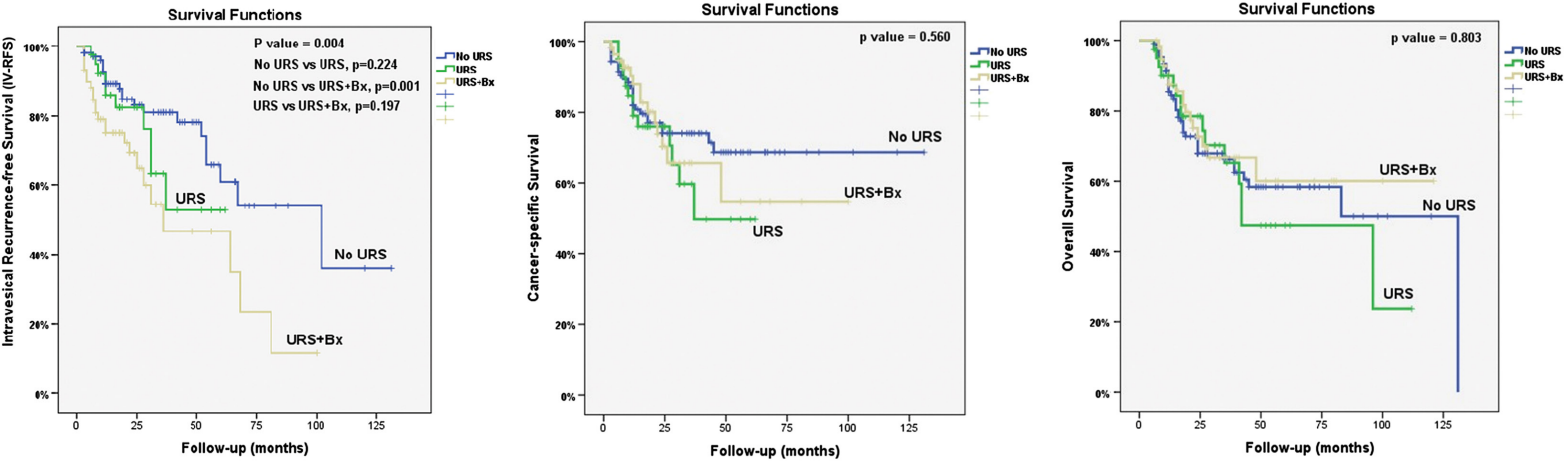
Kaplan–Meier curves for intravesical recurrence-free survival, cancer-specific survival, and overall survival according to groups. URS: ureterorenoscopy, bx: biopsy.

In oncologic follow-up, 22 (20.8%) patients in group 1, 10 (24.4%) patients in group 2, and 23 (39%) patients in group 3 experienced IVR (*P* = .037). The 2-year IV-RFS rate by Kaplan–Meier estimation was 83.1%, 82.4%, and 69.2% for groups 1, 2, and 3, respectively (*P* = .004). According to these analyses, patients with d-URS + bx before RNU had lower IV-RFS than patients with no URS before RNU (*P* = .001) (Figure 2). However, IV-RFS was comparable in groups 1 and 2 (*P* = .224) and also in group 2 and 3 (*P* = .197). During the follow-up, 21 (19.8%), 10 (24.4%), and 14 (23.7%) patients progressed to metastatic diseases in group 1, 2, and 3, respectively (*P* = .765). In the Kaplan–Meier analysis, CSS and OS were similar between the 3 groups (*P* = .560 and *P* = .803, respectively) (Figure 2).

**Figure 3.**
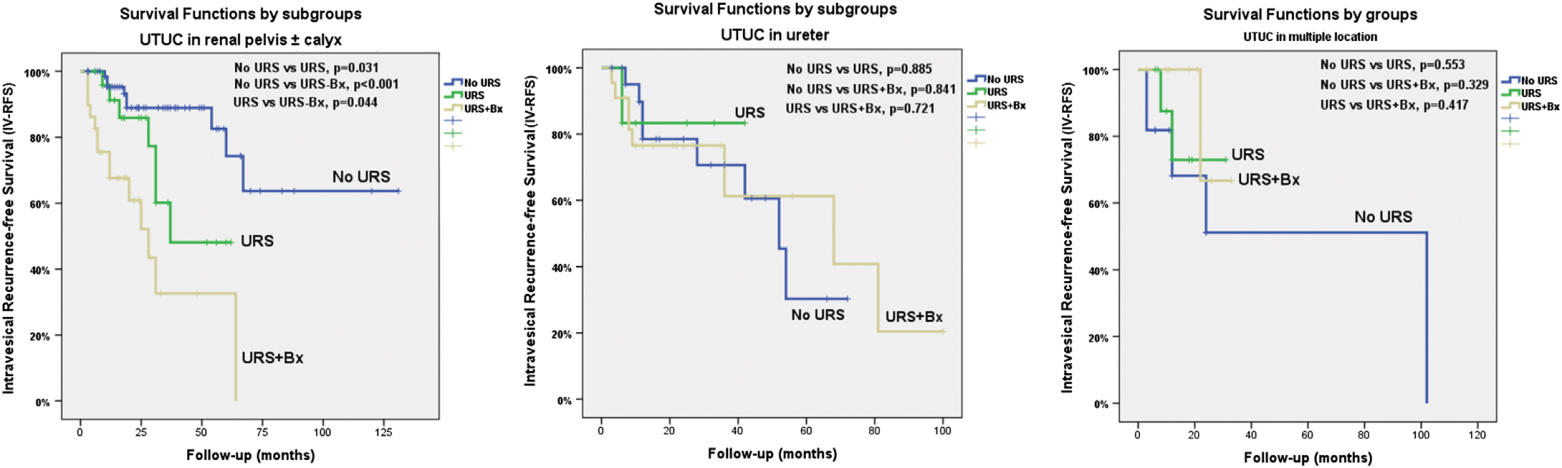
Kaplan–Meier curves for intravesical recurrence-free survival according to tumor location. URS: ureterorenoscopy, bx: biopsy, UTUC: upper tract urothelial carcinoma.

In the subgroup analysis, according to tumor location, in patients with renal pelvic and/or calyceal tumors, d-URS + bx was associated with lower IV-RFS than no URS (*P* < .001) and d-URS (*P* = .044). In addition to this finding, d-URS was associated with lower IV-RFS than no URS (*P* = .031). However, in patients with ureteral or multiple-located (kidney and ureteral) tumors, the difference in IV-RFS across the 3 groups was not statistically significant (Figure 3).

In the multivariate Cox regression analysis, d-URS + bx [hazard ratio (HR): 2.38, 95% CI: 1.29-4.37, *P* = .005] and multifocal tumor (HR: 1.98, 95% CI: 1.02-3.83, *P* = .042) were the independent predictors of IVR after RNU. Ureterorenoscopy without biopsy (HR: 1.36, 95% CI: 0.61-2.99, *P* = .442) was not an independent predictor of IVR (Table 2). After stratification of the patients according to tumor location, it was revealed that d-URS + bx (HR: 6.88, 95% CI: 2.41-19.65, *P* < .001) was the only independent predictor of IVR in patients with UTUC located in the kidney. For ureteral tumors, the only predictive factor was the presence of decreased estimated glomerular filtration rates (eGFRs) (<60 mL/min). Ureterorenoscopy, with or without biopsy, was not a predictive factor for IVR in ureteral tumors. Finally, there was no independent predictor for IVR in patients with tumors located in both the kidney and the ureter (Table 3).

## Discussion

In this retrospective multicenter study, d-URS + bx and tumor multifocality were the independent predictors of IVR in patients with UTUC underwent RNU. When tumor location stratified as kidney, ureter, or multifocal, it was revealed that d-URS + bx was the only predictor of IVR in tumors located to the kidney, and decreased eGFR (<60 mL/min) was the only predictor of IVR in tumors located to the ureter.

Intravesical recurrence is a fairly common problem after RNU and can cause serious morbidity. The most widely accepted theory about the cause of IVR is “intraluminal tumor seeding.” In a previous study, the authors demonstrated that bladder carcinoma cells could grow on repaired mucosa if it is injured by acid or scalpel.^10^ This study supports the theory that free circulating tumor cells in urine can implant to the damaged bladder mucosa and cause IVR. Therefore, d-URS before RNU may increase the spillage of free tumor cells in urine while taking a biopsy, making irrigation, or manipulation such as laser ablation, and may increase IVR.

To date, there are several studies aiming to evaluate factors that predict IVR after RNU. In a recent systematic review and meta-analysis, male gender, extravesical bladder cuff removal, positive surgical margin, laparoscopic technique, necrosis, invasive stage, ureteral location, multifocality, preoperative positive urine cytology, preoperative chronic kidney disease, and history of bladder cancer were reported as independent predictors of IVR after RNU.^4^ In this study, we found that multifocal tumor and d-URS with tumor biopsy were independent predictors of IVR. Multifocal tumor is a well-known predictor of IVR, and these findings are supported by several studies.^11,12^ Hirano et al^12^ investigated the factors that increased the likelihood of after RNU. They included 151 patients without a history of bladder cancer and concluded that tumor multifocality was the only independent predictor of IVR (risk ratio (RR): 4.024, *P* = .001). Two main potential hypotheses have been proposed for the pathophysiology of multifocal urothelial cancers. Tumors that spread throughout the urothelium (via intraluminal seeding or intraepithelial migration) and have a single genetic origin are described under the monoclonality theory. The second hypothesis, that of field cancerization, describes the emergence of synchronous tumors after exposure of the entire urothelium to carcinogens.^13^ These 2 hypotheses identifying the causes of multifocal tumors may also explain the increased IVR in multifocal tumors.

Ureterorenoscopy is commonly used to verify the diagnosis of UTUC for indeterminate lesions. However, the probability of intraluminal tumor seeding by irrigation or manipulation led to a suspicion of increased IVR. In this study, we found that d-URS + bx was an independent predictor of IVR but URS without biopsy was not. Several studies have investigated the impact of d-URS before RNU on IVR and concluded that URS before RNU was linked to a higher IVR.^6,14-16^ However, there are only a few studies that stratify patients as with or without biopsy. In the most recent study, Sharma et al^9^ investigated the impact of the diagnostic modalities of UTUC on IVR after RNU and classified patients by diagnostic modality: no URS or percutaneous biopsy, URS without biopsy, percutaneous biopsy, and URS with biopsy. Similar to our findings, they concluded that URS + bx was associated with increased IVR but not URS without biopsy. However, there are also conflicting results on this issue. In a previous study, Ishikawa et al^17^ reported that d-URS, with or without biopsy, did not affect IVR after RNU.

After stratification of the patients by tumor location, we found that increased IVR rates due to d-URS + bx were only valid for patients with a tumor located in the kidney. In the only previous study that stratified patients by tumor location and evaluated the effect of URS + bx, the authors included 387 patients and reported that a history of bladder cancer was the only significant predictor of IVR in all patients, and URS + bx was the only risk factor for IVR for tumors located in the kidney.^18^ In that study, they did not examine the effect of d-URS without biopsy and included patients with a history of bladder cancer, unlike our study. We excluded these patients because it is well known that the presence of previous bladder cancer is a strong predictor of IVR.

In previous studies, it is demonstrated that ureteral tumor location independently increased the rates of IVR.^4^ This increase could be due to the anatomical proximity of primary tumor to the bladder and increased tumor spillage to the bladder. Diagnostic ureterorenoscopy with biopsy might not further increase the tumor spillage in ureteral tumors which is already increased by anatomical proximity. However, in tumors that are located in renal pelvis, manipulations with URS might increase the tumor spillage, with these tumor fragments reaching the bladder. Diagnostic ureterorenoscopy might eliminate the protective status of anatomical locations of renal-located tumors.

In addition to these findings, we found that decreased eGFR (<60 mL/min) was associated with a higher IVR in tumors located in the ureter. This finding was in line with earlier researches published by Chung et al^19^ and Li et al.^20^ It is reported that patients with chronic kidney diseases have a high frequency of urothelial cancer, and some of the potential mechanisms of carcinogenesis in these patients are chronic inflammation, accumulation of carcinogenic metabolites, reduced antioxidant defense, and impaired function of the immune system.^21,22^

Despite higher IVR with d-URS + bx, CSS or OS was similar in all the groups (no URS, d-URS, or d-URS + bx) in our study, in accordance with previous studies.^7,23^ Intravesical recurrence after RNU are mostly non-muscle invasive and can be managed with a transurethral resection of the bladder tumor and intravesical treatment.^18^ Another concern about CSS and OS is the delay time for RNU because of preoperative d-URS. Boorjian et al^24^ investigated the effect of a delay to RNU for patients undergoing d-URS + bx and ablation of UTUC and included 121 patients in their study. The mean delay time from d-URS + bx to RNU was 28 days and from d-URS + bx and laser ablation to RNU was 196 days. In conclusion, the authors stated that there was no significant difference between the groups according to the postoperative disease status. Similarly to these findings, in our study, treatment delay due to the d-URS + bx was not a significant predictor of IVR.

After studies reported that d-URS was associated with increased IVR, some protective measures have been introduced. In a recent study of IVR after RNU, Baboudjian et al^25^ investigated the effects of technical precautions to avoid tumor spread and contact of tumor cells with bladder mucosa, such as the routine use of ureteral access sheaths, f-ureteroscopy, and mono-J catheters. The authors reported that patients with d-URS had high IVR despite technical precautions. Post d-URS single-dose intravesical chemotherapy administration may be another protective measure for IVR. It is known that single-dose intravesical mitomycin-C administration after RNU decreased IVR, and this method was recommended by European Association of Urology (EAU) guidelines.^2,26^ To date, there has been no study investigating the effect of post d-URS single-dose intravesical mitomycin-C administration for UTUC on IVR. The need for randomized controlled studies on this issue is obvious. Percutaneous biopsy could be performed to prevent increased IVR rates due to d-URS. Despite some concerns about tract seeding, recent reports demonstrated that percutaneous biopsy with coaxial technique could be used effectively for diagnosis of UTUC without compromising short-term oncologic results.^27,28^ Last, another way to make a diagnosis without bx may be to take a barbotage cytology. In the literature, it has been shown that the diagnostic value of barbotage cytology can be high even in low-grade cancers and superior to voiding cytology.^29,30^ In this study, we investigated the effect of d-URS, with and without biopsy, on IVR, with stratification according to tumor location in a homogeneous, multicenter cohort. However, our study had some limitations. Most important limitations were the retrospective design, the long time period for the included patient cohort, and multiple surgeons with unknown surgical experience. The decision to perform d-URS, with or without biopsy, was taken by the surgeon of the patients, and there is no standardized approach for this issue. In addition, we had no information about the technical details of the d-URS, such as the use of access sheaths, flexible ureteroscopes, or technical precautions to avoid tumor spillage. Despite these limitations, our study was the first study that stratified patients by both tumor location and biopsy status. However, it is obvious that further prospective trials are required to provide strong recommendations about this issue.

In patients with UTUC, d-URS + bx before RNU was substantially related to higher IVR, but d-URS without biopsy was not. After stratification of the patients by tumor location, we found that increased IVR rates with d-URS + bx were only valid for tumors located in the kidney. For tumors located in the ureter, the only predictive factor for IVR was eGFR < 60 mL/min. Despite high IVR with d-URS + bx, CSS and OS were similar between the groups with no URS, d-URS, and d-URS + bx.

To conclude, while d-URS + bx before RNU significantly increased the rates of IVR in tumors located in renal pelvis or calyces, it did not increase IVR rates in ureteral or multiple-located tumors. Only d-URS without bx did not increase IVR rates. Also, d-URS + bx did not affect CSS and OS. It is obvious that more studies focusing on the prevention of intravesical recurrence are needed. In this context, safety of percutaneous biopsy or efficacy of intravesical chemotherapy after d-URS could be studied.

## Figures and Tables

**Table 1.** Distributions of Diagnostic Characteristics According to Groups

	Group 1 (No URS) (n = 106)	Group 2 (d-URS) (n = 41)	Group 3 (d-URS + bx) (n = 59)	*P*
Age, years (mean ± SD)	65.7 ± 10.3	64.4 ± 8.2	62.5 ± 11.7	.164^a^
Gender [n (%)]				.699^b^
Female	24 (22.6)	12 (29.3)	15 (25.4)	
Male	82 (77.4)	29 (70.7)	44 (74.6)	
BMI (mean ± SD)	26.9 ± 4.1	27.4 ± 4.6	27.2 ± 4.2	.819^a^
CCI [median (range)]	4 (0-11)	4 (1-8)	4 (0-7)	.547^c^
Smoking, pack years [median (range)]	11 (0-100)	12 (0-60)	10 (0-80)	.878^c^
Smoking [n (%)]				.829^b^
Never	43 (40.5)	13 (31.7)	25 (42.4)	
Former	27 (25.5)	13 (31.7)	14 (23.7)	
Current	36 (34.0)	15 (36.6)	20 (33.9)	
Initial eGFR, mL/min (mean ± SD)	74.3 ± 21.3	76.4 ± 18.6	75.8 ± 25.1	.838^a^
>60mL/min, n (%)	81 (76.4)	34 (82.9)	44 (74.6)	.597^b^
Hydronephrosis, n (%)				.065^b^
No	21 (19.8)	11 (26.8)	9 (15.3)	
Grade 1	13 (12.3)	5 (12.2)	10 (16.9)	
Grade 2	37 (34.9)	8 (19.5)	22 (37.3)	
Grade 3	14 (13.2)	13 (31.7)	13 (22)	
Grade 4	21 (19.8)	4 (9.8)	5 (8.5)	
Diagnosis treatment period, days [median (range)]	32.5 (6-117)	42 (2-100)	58 (9-115)	.001^c,*^
Multifocal tumor, n (%)	14 (13.2)	14 (34.1)	15 (25.4)	.012^b^^,^ **^†^**
Tumor size, mm [median (range)]	50 (3-160)	40 (15-160)	38 (4-120)	.001^c^ **^,^** ^‡^
Location of tumor, n (%)				.013**^b^**
Kidney	73 (68.9)	25 (61)	29 (49.2)	
Ureter	22 (20.8)	6 (14.6)	22 (37.3)	
Kidney + ureter	11 (10.4)	10 (24.4)	8 (13.6)	
pT stage, n (%)				.137^d^
pTa	25 (23.6)	7 (17.1)	13 (22)	
pT1	28 (26.4)	7 (17.1)	18 (30.5)	
pT2	9 (8.5)	3 (7.3)	8 (13.6)	
pT3	32 (30.2)	21 (51.2)	17 (28.8)	
pT4	12 (11.3)	3 (7.3)	3 (5.1)	
Presence of CIS, n (%)	13 (12.3)	6 (14.6)	10 (16.9)	.704^b^
Histological grade, n (%)				.093^b^
Low grade	35 (33)	7 (17.1)	13 (22)	
High grade	71 (67)	34 (82.9)	46 (78)	
pN stage, n (%)				.100^b^
cN0	90 (84.9)	40 (97.6)	52 (88.1)	
cN1-3	16 (15.1)	1 (2.4)	7 (11.9)	
Surgical technique, n (%)				.564^d^
Open	68 (64.2)	21 (51.2)	36 (61)	
Laparoscopic	35 (33)	19 (46.4)	23 (39)	
Robotic	3 (2.8)	1 (2.4)	0	
Complications, n (%)				.983^d^
Grade 0-2	98 (92.5)	38 (92.7)	55 (93.2)	
Grade 3-5	8 (7.5)	3 (7.3)	4 (6.8)	
Adjuvant chemotherapy, n (%)	25 (23.6)	14 (34.1)	14 (23.7)	.387^b^
Intravesical recurrence, n (%)	22 (20.8)	10 (24.4)	23 (39)	.037**^b^**
Metastasis in follow-up, n (%)	21 (19.8)	10 (24.4)	14 (23.7)	.765^b^
Follow-up status, n (%)				.470^b^
Alive	68 (64.2)	26 (63.4)	42 (71.2)	
Cancer-specific death	26 (24.5)	13 (31.7)	14 (23.7)	
Non-cancer related death	12 (11.3)	2 (4.9)	3 (5.1)	
Follow-up, months [median (range)]	24 (6-131)	25 (6-112)	24 (7-121)	.720^c^

^*^Group 1 vs. group 2, *P* = .603^e^; group 1 vs. group 3, *P* < .001^e^; group 2 vs. group 3, *P* = .019^e^.

^†^Group 1 vs. group 2, *P* = .004^b^; group 1 vs. group 3, *P* = .048^b^; group 2 vs. group 3, *P* = .344^b^.

^‡^Group 1 vs. group 2, *P* = .021^e^; group 1 vs. group 3, *P* = .001^e^; group 2 vs. group 3, *P* = .495^e^.

^a^One-way analysis of variance (ANOVA) test,^ b^Pearson chi-square test, ^c^Kruskal–Wallis test, ^d^Fisher’s exact test, ^e^Mann–Whitney *U*-test.

BMI: body mass index, CCI: Charlson comorbidity index, CIS: carcinoma in situ, eGFR: estimated glomerular filtration rate, URS: ureterorenoscopy, d-URS: diagnostic ureterorenoscopy, d-URS+bx: diagnostic ureterorenoscopy+biopsy.

**Table 2.** Univariate and Multivariate Cox Proportional Hazards Regression Analysis to Determine the Factors That Were Associated with Intravesical Recurrence

	Univariate		Multivariate	
	HR (95% CI)	*P*	HR (95% CI)	*P*
	Total (n = 206)			
Age	0.995 (0.969-1.022)	.729		
Gender (male vs. female)	0.926 (0.494-1.736)	.809		
Smoking, pack years	1.005 (0.993-1.017)	.429		
Initial eGFR > 60 mL/min (yes vs. no)	0.788 (0.433-1.436)	.437		
Diagnosis treatment period	1.008 (0.998-1.018)	.106		
Preoperative URS (ref.: no URS)				
d-URS	1.610 (0.751-3.454)	.221	1.362 (0.619-2.994)	.442
d-URS + bx	2.625 (1.446-4.768)	.002	2.382 (1.296-4.378)	.005
Tumor location (ref.: kidney)				
Ureter	1.456 (0.801-2.645)	.217		
Kidney + ureter	1.697 (0.769-3.745)	.191		
Presence of CIS (yes vs. no)	1.572 (0.808-3.061)	.183		
pT stage (pT2-4 vs. pTa-1)	0.914 (0.749-1.116)	.378		
Histological grade (high vs. low)	1.150 (0.642-2.060)	.638		
Multifocal tumor (yes vs. no)	2.321 (1.227-4.390)	.010	1.983 (1.026-3.832)	.042
Tumor size	0.998 (0.986-1.009)	.661		
Lymph node positivity (yes vs. no)	0.361 (0.107-1.224)	.102	0.260 (0.063-1.083)	.064
Surgical technique (min inv vs. open)	1.179 (0.814-1.708)	.384		
Adjuvant treatment (yes vs. no)	0.972 (0.500-1.889)	.932		

CIS: carcinoma in situ, d-URS: diagnostic ureterorenoscopy, d-URS+bx: diagnostic ureterorenoscopy+biopsy, eGFR: estimated glomerular filtration rate, HR: hazard ratio, min inv: minimally invasive, URS: ureterorenoscopy

**Table 3.** Univariate and Multivariate Cox Proportional Hazards Regression Analysis to Determine the Factors That Were Associated with Intravesical Recurrence-Free Survival According to the Tumor Location

	Univariate		Multivariate		Univariate		Multivariate		Univariate		Multivariate	
	HR (95% CI)	*P*	HR (95% CI)	*P*	HR (95% CI)	*P*	HR (95% CI)	*P*	HR (95% CI)	*P*	HR (95% CI)	*P*
	Kidney (n = 127)				Ureter (n = 50)				Kidney + Ureter (n = 29)			
Age	0.988 (0.955-1.022)	.492			1.008 (0.956-1.062)	.780			1.021 (0.939-1.110)	.634		
Gender (male vs. female)	1.691 (0.645-4.434)	.286			0.633 (0.198-2.027)	.441			0.283 (0.062-1.297)	.104		
Smoking. pack years	1.009 (0.994-1.023)	.231			1.015 (0.986-1.044)	.312			0.987 (0.939-1.038)	.611		
Initial eGFR > 60mL/min (yes vs. no)	0.973 (0.417-2.271)	.950			0.298 (0.101-0.879)	.028	0.299 (0.101-0.882)	0.029	1.386 (0.268-7.177)	.697		
Diagnosis treatment period	1.005 (0.992-1.017)	.463			1.008 (0.990-1.026)	.383			1.021 (0.987-1.055)	.224		
Preoperative URS (ref.: No URS)												
d-URS	2.977 (1.067-8.309)	.037	2.702 (0.916-7.969)	0.072	0.721 (0.089-5.844)	.759			0.693 (0.121-3.969)	.681		
d-URS + bx	7.540 (3.101-18.332)	<.001	6.883 (2.410-19.654)	<0.001	0.896 (0.320-2.510)	.834			0.334 (0.037-3.007)	.328		
Presence of CIS (yes vs. no)	1.038 (0.358-3.010)	.946			1.839 (0.516-6.556)	.347			3.129 (0.678-14.448)	.144	3.853 (0.781-19.006)	.098
pT stage (pT2-4 vs. pTa-1)	0.554 (0.263-1.167)	.120			1.140 (0.418-3.112)	.798			1.505 (0.281-8.060)	.633		
Histological grade (high vs. low)	1.120 (0.510-2.460)	.777			0.799 (0.296-2.155)	.658			24.067 (0.001-1 011 509.514)	.558		
Multifocal tumor (yes vs. no)	3.118 (1.173-8.294)	.023	1.755 (0.611-5.039)	0.296	1.737 (0.383-7.867)	.474			0.972 (0.405-2.337)	.950		
Tumor size	0.993 (0.976-1.010)	.399			1.005 (0.985-1.025)	.648			0.999 (0.979-1.020)	.951		
Lymph node positivity (yes vs. no)	0.330 (0.045-2.427)	.276			0.031 (0.000-19.697)	.291			0.850 (0.102-7.077)	.880		
Surgical technique (min inv vs. open)	1.170 (0.711-1.925)	.536			1.078 (0.517-2.247)	.841			1.672 (0.684-4.091)	.260		
Adjuvant treatment (yes vs. no)	0.562 (0.196-1.616)	.285			0.846 (0.189-3.791)	.827			4.098 (0.785-21.397)	.094	4.911 (0.894-26.981)	.067

CIS: carcinoma in situ, d-URS: diagnostic ureterorenoscopy, d-URS+bx: diagnostic ureterorenoscopy+biopsy, eGFR: estimated glomerular filtration rate, HR: hazard ratio, min inv: minimally invasive, URS: ureterorenoscopy.

## References

[1] Soria F Shariat SF Lerner SP et al. Epidemiology, diagnosis, preoperative evaluation and prognostic assessment of upper-tract urothelial carcinoma (UTUC). World J Urol. 2017;35(3):379 387. 10.1007/s00345-016-1928-x) 27604375

[2] Rouprêt M Babjuk M Burger M et al. European Association of Urology guidelines on upper urinary tract urothelial carcinoma: 2020 update. Eur Urol. 2020;9:80 81. 10.1016/j.eururo.2020.05.042) 32593530

[3] Margulis V Shariat SF Matin SF et al. Outcomes of radical nephroureterectomy: a series from the upper tract urothelial carcinoma collaboration. Cancer. 2009;115(6):1224 1233. 10.1002/cncr.24135) 19156917

[4] Seisen T Granger B Colin P et al. A systematic review and meta-analysis of clinicopathologic factors linked to intravesical recurrence after radical nephroureterectomy to treat upper tract urothelial carcinoma. Eur Urol. 2015;67(6):1122 1133. 10.1016/j.eururo.2014.11.035) 25488681

[5] Hendin BN Streem SB Levin HS Klein EA Novick AC . Impact of diagnostic ureteroscopy on long-term survival in patients with upper tract transitional cell carcinoma. J Urol. 1999;161(3):783 785. 10.1016/S0022-5347(01)61768-3) 10022684

[6] Liu P Su XH Xiong GY Li XS Zhou LQ . Diagnostic ureteroscopy for upper tract urothelial carcinoma is independently associated with intravesical recurrence after radical nephroureterectomy. Int Braz J Urol. 2016;42(6):1129 1135. 10.1590/S1677-5538.IBJU.2015.0366) 27509369 PMC5117968

[7] Lee HY Yeh HC Wu WJ et al. The diagnostic ureteroscopy before radical nephroureterectomy in upper urinary tract urothelial carcinoma is not associated with higher intravesical recurrence. World J Surg Oncol. 2018;16(1):135. 10.1186/s12957-018-1411-9) 29986730 PMC6038188

[8] Sankin A Tin AL Mano R et al. Impact of ureteroscopy before nephroureterectomy for upper tract urothelial carcinoma on oncologic outcomes. Urology. 2016;94:148 153. 10.1016/j.urology.2016.05.039) 27237781 PMC5114126

[9] Sharma V Miest TS Juvet TS et al. The impact of upper tract urothelial carcinoma diagnostic modality on intravesical recurrence after radical nephroureterectomy: a single institution series and updated meta-analysis. J Urol. 2021;206(3):558 567. 10.1097/JU.0000000000001834) 33908802

[10] Nakamura K Fujiyama C Tokuda Y Sugihara H Masaki Z . Bladder cancer cell implantation in reconstructed bladder in vitro: a model of tumour recurrence. BJU Int. 2002;89(1):119 125. 10.1046/j.1464-4096.2001.01642.x) 11849176

[11] Xylinas E Colin P Audenet F et al. Intravesical recurrence after radical nephroureterectomy for upper tract urothelial carcinomas: predictors and impact on subsequent oncological outcomes from a national multicenter study. World J Urol. 2013;31(1):61 68. 10.1007/s00345-012-0957-3) 23053211

[12] Hirano D Okada Y Nagane Y et al. Intravesical recurrence after surgical management of urothelial carcinoma of the upper urinary tract. Urol Int. 2012;89(1):71 77. 10.1159/000338644) 22677699

[13] Hafner C Knuechel R Stoehr R Hartmann A . Clonality of multifocal urothelial carcinomas: 10 years of molecular genetic studies. Int J Cancer. 2002;101(1):1 6. 10.1002/ijc.10544) 12209580

[14] Chung Y Lee DH Lee M et al. Impact of diagnostic ureteroscopy before radical nephroureterectomy on intravesical recurrence in patients with upper tract urothelial cancer. Investig Clin Urol. 2020;61(2):158 165. 10.4111/icu.2020.61.2.158) PMC7052419 32158966

[15] İzol V Deger M Ozden E et al. The effect of diagnostic ureterorenoscopy on intravesical recurrence in patients undergoing nephroureterectomy for primary upper tract urinary carcinoma. Urol Int. 2021;105(3-4):291 297. 10.1159/000511650) 33264798

[16] Sung HH Jeon HG Han DH et al. Diagnostic ureterorenoscopy is associated with increased intravesical recurrence following radical nephroureterectomy in upper tract urothelial Carcinoma. PLoS One. 2015;10(11):e0139976. 10.1371/journal.pone.0139976) 26556239 PMC4640521

[17] Ishikawa S Abe T Shinohara N et al. Impact of diagnostic ureteroscopy on intravesical recurrence and survival in patients with urothelial carcinoma of the upper urinary tract. J Urol. 2010;184(3):883 887. 10.1016/j.juro.2010.05.027) 20643446

[18] Yoo S You D Song C et al. Risk of intravesical recurrence after ureteroscopic biopsy for upper tract urothelial carcinoma: does the location matter? J Endourol. 2017;31(3):259 265. 10.1089/end.2016.0611) 27785917

[19] Chung SD Huang KH Lai MK et al. CKD as a risk factor for bladder recurrence after nephroureterectomy for upper urinary tract urothelial carcinoma. Am J Kidney Dis. 2007;50(5):743 753. 10.1053/j.ajkd.2007.08.007) 17954287

[20] Li CC Chang TH Wu WJ et al. Significant predictive factors for prognosis of primary upper urinary tract cancer after radical nephroureterectomy in Taiwanese patients. Eur Urol. 2008;54(5):1127 1134. 10.1016/j.eururo.2008.01.054) 18243511

[21] Chen KS Lai MK Huang CC Chu SH Leu ML . Urologic cancers in uremic patients. Am J Kidney Dis. 1995;25(5):694 700. 10.1016/0272-6386(95)90544-8) 7747722

[22] Vamvakas S Bahner U Heidland A . Cancer in End-Stage Renal Disease: potential factors involved -editorial-. Am J Nephrol. 1998;18(2):89 95. 10.1159/000013314) 9569948

[23] Luo HL Kang CH Chen YT et al. Diagnostic ureteroscopy independently correlates with intravesical recurrence after nephroureterectomy for upper urinary tract urothelial carcinoma. Ann Surg Oncol. 2013;20(9):3121 3126. 10.1245/s10434-013-3000-z) 23661184

[24] Boorjian S Ng C Munver R et al. Impact of delay to nephroureterectomy for patients undergoing ureteroscopic biopsy and laser tumor ablation of upper tract transitional cell carcinoma. Urology. 2005;66(2):283 287. 10.1016/j.urology.2005.02.022) 16098357

[25] Baboudjian M Al-Balushi K Michel F et al. Diagnostic ureteroscopy prior to nephroureterectomy for urothelial carcinoma is associated with a high risk of bladder recurrence despite technical precautions to avoid tumor spillage. World J Urol. 2020;38(1):159 165. 10.1007/s00345-019-02768-w) 30993427

[26] O’Brien T Ray E Singh R Coker B Beard R . Prevention of bladder tumours after nephroureterectomy for primary upper urinary tract urothelial carcinoma: a prospective, multicentre, randomised clinical trial of a single postoperative intravesical dose of Mitomycin C (the ODMIT-C Trial). Eur Urol. 2011;60(4):703 710. 10.1016/j.eururo.2011.05.064) 21684068

[27] Joseph JP Potretzke TA Packiam V et al. Percutaneous image-guided core needle biopsy for upper tract urothelial carcinoma. Urology. 2020;135:95 100. 10.1016/j.urology.2019.10.005) 31655078

[28] Huang SY Ahrar K Gupta S et al. Safety and diagnostic accuracy of percutaneous biopsy in upper tract urothelial carcinoma. BJU Int. 2015;115(4):625 632. 10.1111/bju.12824) 24905868

[29] Zhang ML Rosenthal DL VandenBussche CJ . Upper urinary tract washings outperform voided urine specimens to detect upper tract high-grade urothelial carcinoma. Diagn Cytopathol. 2017;45(8):700 704. 10.1002/dc.23746) 28556525

[30] Malm C Grahn A Jaremko G Tribukait B Brehmer M . Diagnostic accuracy of upper tract urothelial carcinoma: how samples are collected matters. Scand J Urol. 2017;51(2):137 145. 10.1080/21681805.2017.1295102) 28385123

